# Myeloperoxidase level and inflammatory markers and lipid and lipoprotein parameters in stable coronary artery disease

**DOI:** 10.1186/s12944-018-0718-4

**Published:** 2018-04-04

**Authors:** Elżbieta Kimak, Bartosz Zięba, Dariusz Duma, Janusz Solski

**Affiliations:** 1 Department of Laboratory Diagnostics, Medical University, Street Chodźki 1, 20-093 Lublin, Poland; 2 Department of Cardiology of the Provincial Specialistics Cardinal Stefan Wyszynski Hospital, Lublin, Poland

**Keywords:** Myeloperoxidase, Inflammatory markers, Lipids, Lipoproteins, Stable coronary

## Abstract

**Background:**

Myeloperoxidase (MPO) impairing endothelial functions. We investigated whether increasing concentration of myeloperoxidase (MPO) and inflammatory markers induce progression and incident acute coronary syndrome (ACS) in stable coronary artery disease (SCAD) patients. Therefore, the concentration of MPO, lipids, lipoproteins (apo(apolipoprotein) AI, apoB, lipoprotein associated phospholipase A2 (LpPLA2) level), inflammatory markers (high sensitivity C-reactive protein (hsCRP), tumor necrosis factor-α (TNF-α), interleukine-6 (IL-6) concentration) were examined.

**Methods:**

This study concerned 67 SCAD patients divided into groups: all patients, patients with MPO < 200 ng/ml, MPO 200–300 ng/ml, MPO > 300 ng/ml concentration and 15 controls. ApoAI, apoB and hsCRP levels were examined using the immunonephelometric method, and MPO, LpPLA2, IL-6, TNF-α concentration was performed by using Quantikine ELISA kit R&D Systems.

**Results:**

In the all patients, and in group with MPO 200–300 ng/ml TC, LDL-C, nonHDL-C, LpPLA2 concentration and TC/HDL-C, LDL-C/HDL-C ratios were insignificant, and significantly higher concentration of TG, apoB, MPO, inflammatory markers and TG/HDL-C, MPO/apoAI, MPO/HDL-C ratios but HDL-C, apoAI level and HDL-C/apoAI ratio were significantly reduced. In the group of patients with MPO < 200 ng/ml, level of TC, LDL-C, nonHDL-C, apoAI, apoAII, LpPLA2 and MPO and LDL-C/HDL-C ratio were in-significant, HDL-C was decreased but apoB, TG, inflammatory markers, apoB/apoAI, TG/HDL-C, MPO/apoAI, MPO/HDL-C ratio were significantly increased. In the group of patients with MPO > 300 ng/ml concentration of TC, LDL-C, nonHDL-C, apoAII, LpPLA2 and LDL-C/HDL-C ratios were not significant, but HDL-C and apoAI concentrations were significantly decreased. The concentrations of TG, apoB, MPO and inflammatory markers and TG/HDL-C, MPO/apoAI, MPO/HDL-C ratios were significantly increased compared to the controls. The apoAI concentration was significantly decreased and the concentration of MPO and hsCRP as well as MPO/apoAI and MPO/HDL-C ratios were significantly higher as compared to the group of patients with MPO < 200 ng/ml.

Spearman’s correlation test showed a positive correlation between MPO concentration and MPO/apoAI and MPO/HDL-C ratios in all patients and MPO < 200 ng/ml, MPO 200–300 ng/ml. The patients with MPO > 300 ng/ml showed a positive correlation between the concentration of MPO and the level of hsCRP and IL-6, and a negative correlation between MPO/apoAI ratio and the concentration of HDL-C, apoAI and apoAII.

**Conclusion:**

The results suggest that moderate dyslipidemia and dyslipoproteinemia deepening of inflammation, and inflammation slowly induce increase MPO concentration which decrease apoAI and HDL-C level and disturb HDLs function. The increasing MPO level and MPO/HDL-C, MPO/apoAI ratios can differentiate the SCAD patients at the risk of acute coronary syndrome (ACAD) and stroke.

## Background

Patients with stable coronary artery disease (SCAD) are a heterogenous group in terms of the progression rate of the disease, the severity of coronary lesions and the occurrence of acute coronary syndromes (ACS) [1–3]. Oxidative stress and inflammation play the direct role in the initial and progression of atherosclerosis plaques and development of cardiovascular artery disease (CAD) [4, 5]. A high density lipoprotein (HDL) protects against coronary artery disease (CAD) mainly by reverse cholesterol transport. Moreover, HDL has anti-inflammatory, anti-oxidative, in part via paraoxonase-1 (PON-1), anti-thrombotic and anti-apoptotic properties, increased endothelial cells (EC), nitric oxide synthase, enhanced endothelial progenitor cells (EPC) mediating vasculoprotection, stimulation of endothelial cell proliferation and migration [6]. Myeloperoxidase (MPO), a product of systemic inflammation plays important role both in the process of oxidative stress and inflammation and promotes oxidation of lipoproteins [5]. MPO is a leukocyte-derived enzyme that catalyzes the formation of oxidative reactants and participates in innate immunity against infections. MPO is a potential participant in promotion and propagation of atherosclerosis [7] and impairing endothelial function, it can participate in destabilization of atherosclerotic plaques [8]. Myeloperoxidase selectively induces HDL oxidation within the artery wall converting anti-atherogenic lipoprotein into potential atherogenic forms [7, 8]. Myeloperoxidase (MPO) concentrations predict adverse clinical outcomes in setting acute coronary syndromes (ACS) and heart failure, but the prognostic role of MPO in SCAD with atherosclerotic burden is poorly understood [9]. Unfortunately, there is little information indicating the influence the MPO and inflammatory markers level on the concentration of apoAI and HDL-C and the stability of atherosclerotic plaques in SCAD patients.

The aim of the study was to investigate whether increasing concentration of myeloperoxidase (MPO) and inflammatory markers induce progression and incident ACS in groups of patients with stable cardiovascular artery disease patients. Therefore, the authors decided to examine the concentration of MPO, lipids, lipoproteins (apo(apolipoprotein)AI, apoB, lipoprotein associated phospholipase A2 (LpPLA2) level), inflammatory markers (high sensitivity C-reactive protein (hsCRP), tumor necrosis factor-α (TNF-α), interleukine-6 (IL-6) concentration) in SCAD patients.

## Methods

### Patients

This study concerned 67 patients with SCAD, 38 females and 29 males, aged 56–70 years, hospitalized in the Department of Cardiology of the Provincial Specialistics Cardinal Stefan Wyszynski Hospital in Lublin, Poland. All patients in this study received acetyl salicylic acid and statin and were treated according to the ESC Guidelines on the treatment of stable coronary artery disease [10]. Patients with acute and chronic renal failure, liver disease, active autoimmune disease, cancer, thyroid disease, alcoholism and smokers and former smokers were excluded from the study. In the group of patients with hypertension were those receiving antihypertensive drugs which did not affect the status of lipid and lipoprotein. Diabetes mellitus was defined in patients under active treatment with insulin or oral hypoglycemic medication. All patients were divided into groups: concentration of MPO < 200 ng/ml, MPO 200–300 ng/ml and MPO > 300 ng/ml. The control group consisted of 15 healthy volunteers with no risk factors for cardiovascular disease, non-smoking, non-taking any medication, and without ischemic heart disease, 10 females and 5 males, aged 27–55 years. The study excluded pregnant women and using hormonal contraception. The qualification basis of volunteers to the control group was normal medical history and laboratory parameters.

### Sample preparation

The material for the study was blood serum of patients with SCAD and healthy people. The patients’ blood serum was collected after 14 h of fasting. For blood serum collection the system of vacuum tubes of sarstedt company was used without anticoagulant. In the fresh serum routine laboratory and lipid parameters were determined. The remainder of the sera was aliquoted, frozen and stored at -80^o^ C.

### Determination of lipids, lipoproteins, hsCRP

Lipids (total cholesterol (TC), LDL-cholesterol (LDL-C), HDL-cholesterol (HDL-C) was determined by routine methods on the Siemens analyzer (Germany), whereas lipoprotein apoAI, apoB, hsCRP by using the immunonephelometric method on BNII Nephelometer BNII (Germany) (Siemens GmbH, Germany). Non-HDL cholesterol (nonHDL-C) was calculated as TC minus HDL-C.

### Determination of MPO, IL-6, TNF-α and LpPLAG7

Determination of IL-6, TNF-α, LpPLA2G7, MPO was performed by using Quantikine ELISA kit R&D Systems, Inc., USA & Canada. The concentration of human IL-6 or TNF-α or LpPLA2G7 or MPO in serum was measured by the sandwich enzyme immunoassay kit. The microplates were pre-coated with monoclonal antibody specific for IL-6 or TNF-α or LpPLA2G7 or MPO. The samples and standards were pipetted into wells in which they were bound by an immobilized antibody. After incubation every microplate was washed with a washing reagent. Then enzyme-linked polyclonal antibody specific for IL-6, or TNF-α or LpPLA2G7 or MPO was added to the wells. After that another incubation amplifier solution was added to the wells. The measured intensity of the developing color was proportional to the concentration of IL-6, or TNF-α or LpPLA2G7 or MPO.

### Statistical analyses

The statistical analyses were performed using STATISTICA software (StatSoft, Krakow, Poland). The normal distribution of the analysed parameters was assessed by the Shapiro-Wilk test. For comparison, of more than two groups the Kruskal-Wallis test was used. The relation between the variables was examined by Spearman’s correlation analysis. The data were expressed as medians (minimum-maximum). The statistical significance of all variables was established at *p* < 0.05.

## Results

The clinical parameters in SCAD patients and controls are shown in Table 1. In all patients diabetes mellitus occurred in 12 patients (18%), and obesity and overweight in 49 patients (73%). Concentration of myeloperoxidase, inflammatory markers and lipid and lipoprotein and lipid and lipoprotein ratios in the patients and the reference group are presented in Table 2. The results showed dyslipidemia and dyslipoproteinemia in SCAD patients. The concentration of lipids and lipoproteins and inflammatory markers of patients were varied and indicated their disturbances. Therefore, groups of all patients, patients with MPO < 200 ng/ml, MPO 200–300 ng ml and MPO > 300 ng/ ml level were separated. The division of patients into groups according to the increasing concentration of MPO caused the selection of patients with deteriorating inflammatory markers which accompanied deteriorating dyslipidemia and dyslipoproteinemia. In the all group of SCAD patients, TC, LDL-C, nonHDL-C, LpPLA2 concentration and TC/HDL-C, LDL-C/HDL-C ratios were statistically insignificant but HDL-C, apoAI level and HDL-C/apoAI ratio were significantly reduced as compared to the controls. In contrast, the concentrations of TG, apoB, inflammatory markers (MPO, hsCRP, TNF-α, IL-6,) and TG/HDL-C, apoB/apoAI, MPO/apoAI and MPO/HDL-C ratios were significantly increased. In the group of patients with MPO < 200 ng/ml, level of TC, LDL-C, nonHDL-C, apoAI, apoAII, LpPLA2 and MPO and LDL-C/HDL-C ratio were in-significantly as compared to controls. However, the concentrations of TG, apoB, hsCRP, TNF-α and IL-6 and apoB/apoAI, MPO/apoAI and MPO/HDL-C ratios were significantly higher than controls. In the group of patients with concentration of MPO 200–300 ng ml, significantly decreased HDL-C and apoAI level and HDL-C/apoAI ratio, and significantly higher concentration of TG, apoB, inflammatory markers (MPO, hsCRP, TNF-α, IL-6 level) and TG/HDL-C, apoB/apoAI, MPO/apoAI, MPO/HDL-C ratios were observed. However, level of TC, LDL-C, nonHDL-C, apoAII, LpPLA2 and TC/HDL-C, LDL-C/HDL-C ratios were in-significant as compared to controls.

**Table 1:** Clinical parameters and the reference group in SCAD patients (median (min-max)

	MPO < 200 ng/ml, *n* = 26	MPO = 200–300 ng/ml *n* = 26	MPO > 300 ng/ml *n* = 15	MPO ng/ml *n* = 67	Controls *n* = 15
Ages (years)	61 (58–67	66 (56–70)	63 (58–68)	63 (56–70)	55 (27–55)
Gender (male/ female)	14 F, 12 M	15F, 11 M	9F, 6 M	38F, 29 M	10F, 5 M
BMI (kg/m^2^)	28.4 (25–30)	27.6 (24.5–29.4)	28.1 (25.1–34.9)	28.0 (24.5–34.9)	25 (22–27)
eGFR (ml/min/1.73 m2)	95.1 (84.4–103.6)	83 (63–94.6)	79 (69–109)	90 (63–109)	125 (95–160)
Creatinine (μmol/L)	87 (77–94)	80 (64–92)	87 (65–97)	80 (72–97)	97 (71–108)
Hypertension	*n* = 9	*n* = 10	*n* = 12	31	–
Diabetes mellitus	–	4	8	12	–

**Table 2:** Myeloperoxidase level, inflammatory markers, lipid, lipoprotein parameters and lipid and lipoprotein ratios in SCAD patients and the reference group (median (minimum-maximum)

	MPO < 200 ng/ml, *n* = 26	MPO200–300 ng/ml *n* = 26	MPO > 300 ng/ml *n* = 15	MPO ng/ml *n* = 67	Controls *n* = 15
TC (mmol/L)	4.50 (3.73–5.02)	3.91 (3.41–4.64)	3.99 (3.03–7.74)	4.33 (3.03–7.74)	4.87 (2.79–5.18)
LDL-C (mmol/L)	2.48 (1.96–3.09)	2.30 (3.95–3.10)	2.20 (1.32–5.49)	2.38 (1.96–5.49)	3.03 (1.10–3.99)
HDL-C (mmol/L)	1.08 (0.93–1.32)**	1.08 (1.01–1.42)**	0.95 (0.60–1.42)***	1.07 (0.60–1.42)**	1.55 (1.14–2.30)
TG (mmol/L)	1.88 (1.58–2.73)*	1.67 (0.93–2.12)*	1.72 (0.93–3.91)*	1.75 (0.93–3.91)*	1.06 (0.34–2.70)
nonHDL-C (mmol/L)	1.51 (1.16–1.71)	1.19 (0.93–1.45)	1.32 (1.07–1.70)	1.40 (0.93–1.71)	1.06 (0.34–2.70)
apoAI (g/L)	1.45 (1.20–1.51)	1.39 (1.29–1.60)*	1.29 (1.15–1.75)**↑	1.38 (1.15–1.75)*	1.57 (1.35–1.99)
apoAII(g/L)	0.29 (0.27–0.32)	0.28 (0.25–0.34)	0.29 (0.25–0.34)	0.29 (0.25–0.34)	0.32 (0.29–0.38)
ApoB (g/L)	0.93 (0.864–1.26)*	0.90 (0.67–1.12)*	0.96 (0.63–1.16)*	0.94 (0.63–1.25)*	0.70 (0.41–1.17)
LpPLA2 (ng/ml)	101 (86–185)	98 (67–164)	112 (79–126)	103 (67–185)	72.4 (32–172.1)
TC/HDL-C	4.15 (3.39–5.36)*	3.59 (1.58–5.80)	4.10 (3.39–4.77)*	3.79 (1.58–5.80)	3.13 (2.60–3.71)
LDL-C/HDL-C	2.26 (2.09–3.13)	2.14 (1.25–2.59)	2.28 (1.76–2.81)	2.25 (1.25–2.81)	1.95 (1.31–2.40)
TG/HDL-C	3.90 (2.04–6.19)*	3.52 (1.79–3.9)*	3.90 (1.65–4.76)*	3.48 (1.79–6.19)*	1.60 (1.12–3.02)
apoB/apoAI	0.62 (0.60–0.90)*	0.65 (0.46–0.84)*	0.75 (0.55–0.86)**	0.69 (0.55–0.90)**	0.46 (0.27–0.64)
HDL-C/apoAI	0.30 (0.27–0.36)**	0.31 (0.29–0.38)**	0.29 (0.26–0.37)**↑	0.31 (0.26–0.38)**	0.39 (0.32–0.46)
MPO ng/ml	138 (72.4–176)	230 (203–263)**	350 (303–393)***	229 (72.4–393)**	90.0 (49–153)
hsCRP (μg/ml)	0.141 (0.086–0.28)**	0.157 (0.086–0.38)**	0.21 (0.086–1.38)***↑	0.152 (0.086–1.38)**	0.09 (0.02–0.5)
TNF-alfa (pg/ml)	5.23 (4.8–8.3)***	5.31 (4.18–6.25)***	5.80 (4.86–6.61)***	5.35 (4.18–8.3)***	1.20 (0.60–1.21)
IL-6 (pg/ml)	1.74 (0.84–3.51)**	2.54 (1.42–3.50)***	3.02 (2.36–3.80)***	2.64 (0.84–3.80)***	0.40 (0.20–0.83)
MPO/apoAI	0.95 (0.61–1.10)*	1.65(1.46–1.88)**	2.72 (2.05–2.92)***↑	1.67 (0.61–2.92)**	O.55 (0.26–2.53)
MPO/HDL-C	2.51 (1.84–4.14)*	5.41 (3.95–5.94)**	8.51 (5.60–9.15)***↑	5.23 (1.84–5.94)**	1.48 (0.63–4.51)

*MPO* myeloperoxidase, *SCAD* stable cardiovascular artery disease

* *P* < 0.05; ** *P* < 0.01; *** *P* < 0.001 vs. the reference group; *↑P* < 0.05 vs. the group of MPO < 200 ng/ml

In the group of patients with MPO > 300 ng/ml concentration of TC, LDL-C, nonHDL-C, apoAII, LpPLA2 and LDL-C/HDL-C ratios were not statistically significant, but HDL-C and apoAI concentration and HDL-C/apoAI were significantly decreased. The concentrations of TG, apoB, MPO and inflammatory markers (MPO, hsCRP, TNF-α, IL-6 level) and TG/HDL-C, apoB/apoAI, MPO/apoAI and MPO/HDL-C ratios were significantly increased compared to the control group. Moreover, the apoAI concentration was significantly decreased and the concentration of MPO and hsCRP as well as MPO/apoAI and MPO/HDL-C ratios were significantly higher as compared to the group of patients with MPO < 200 ng/ml.

Levels of TC, LDL-C and TC/HDL-C and LDL-C/HDL-C ratios were decreased and were dependent on statin treatment. However, statins therapy reduced TG level at a small degree and did not increase HDL-C and apoAI levels. Moreover, concentration of triglyceride rich-lipoproteins (TRLs) and cholesterol-rich lipoproteins (apoB-containing lipoproteins) were increased as TG and apoB concentration and TG/HDL ratio showed.

Correlations were calculated for each group separately. In all patients a significant positive correlation were observed between concentration of MPO and apoAI (*R* = 0.917; *p* = 0.000) and between MPO and MPO/HDL-C (*R* = 0.838; *p* = 0.000).

In patients with MPO < 200 ng/ml a significant positive correlation occurred between the concentration of MPO and MPO/apoAI ratio (*R* = 0.853, *p* = 0.00001) and a significant positive correlation between MPO level and MPO/HDL-C ratio (*R* = 0.729, *p* = 0.00002). In patients with MPO 200–300 ng/ml level a significant positive correlation between MPO concentration and MPO/apoAI ratio (*R* = 0.548, *p* = 0.003) and between MPO level and MPO/HDL-C ratio (*r* = 0.428, *p* = 0.029) was observed. However, in patients with MPO > 300 ng/ml level a significant positive correlation between MPO level and hsCRP concentration (*R* = 0.719, *p* = 0.0044), between MPO level and IL-6 concentration (*R* = 0.857, *p* = 0.0065), and a significant negative correlation between MPO/apoAI ratio and apoAI level (*R* = − 0.758, *p* = 0.0026), between MPO/apoAI ratio and apoAII level (*R* = − 0.764, *p* = 0.0023) and between MPO/apoAI ratio and HDL-C concentration (*R* = − 0.670, *p* = 0.012) was noted.

## Discussion

Coronary artery disease, including its chronic form - stable coronary artery disease, is an example of the clinical condition which progress and development depend on the co-existence of the risk factors [1–4]. Oxidative modification of HDL particles by MPO may contribute to the development of progression atherosclerosis in stable coronary artery disease [5]. In our studies smokers and former smokers with SCAD were excluded, and the results of the patients were compared to non-smoking control [11]. All SCAD patients were treated with statin and acetyl salicylic acid according to the ESC Guidelines on the treatment of stable coronary artery disease [10]. However, in all our patients the lipid and lipoprotein levels varied from low to high, suggesting dyslipidemia and dyslipoproteinemia of the study subjects despite an unquestioned concentration of TC and LDL-C. They had significantly reduced HDL-C and apoAI but increased concentration TG, apoB (apoB-containing lipoproteins triglyceride–rich lipoproteins (TRLs)), MPO and inflammatory markers (hsCRP, TNF-α, IL-6) level. For these reasons it was decided to divide patients into groups in which the concentration of MPO was the factor differentiating SCAD patients. The division of patients into groups according to the increasing concentration of MPO caused the selection of patients along with deteriorating inflammatory markers and worsening dyslipidemia and dyslipoproteinemia. In the group of patients with MPO concentration < 200 ng/ml, decrease HDL-C and apoAI concentration was accompanied by an increase TG, apoB and inflammatory markers but MPO level was insignificant. However, MPO/apoAI and MPO/HDL-C ratios were moderately elevated. These results suggest that dyslipidemia and dyslipoproteinemia deepening of inflammation in SCAD patients. In the group of patients with MPO200–300 ng/ml higher MPO concentration caused deterioration of apoAI concentration, increased levels of markers of inflammation and a significant increase MPO/apoAI and MPO/HDL-C ratios. In the group of patients with concentration of MPO > 300 ng/ml, apoAI, inflammatory markers (MPO, hsCRP, TNF-α, IL-6) level and MPO/apoAI and MPO/HDL-C were significantly worsened, and were significantly higher in comparison to controls and to MPO < 200 ng/ml SCAD patients. These results suggest that increase of inflammation (MPO) deepening of dyslipidemia and dyslipoproteinemia of patients SCAD. In the group of all patients and patients with MPO < 200 ng/ml and with MPO 200–300 ng/dl positive correlations was found between MPO and MPO/apoAI ratio and between MPO and MPO/HDL-C ratio. These positive correlations suggest that a moderately elevated inflammation can cause an increase MPO/apoAI and MPO/HDL-C ratios [12]. Positive correlations between MPO level and hsCRP concentration as well as between MPO concentration and IL-6 level in patients with MPO > 300 ng/ml suggest that increasing levels of inflammatory markers may cause an increase in MPO concentration. However, significant negative correlations between MPO/apoAI ratio and apoAI, apoAII and HDL-C levels suggest that MPO concentration may significantly decrease apoAI, apoAII and HDL-C levels. These results showed that the increase of inflammation (MPO) deepening of dyslipidemia and dyslipoproteinemia of SCAD patients [12, 13].

The concentration of Lp PLA2 was non-statistically increased in our SCAD patients [14]. LpPLA2 is a marker of inflammation and is a link between lipid metabolism and low-grade inflammation characteristic of cardiovascular or metabolic syndrome [15, 16]. The concentration of LpPLA2 is characterized by minimal biological variability and high specificity for vasculitis and is non-depending from insulin resistance [17]. Therefore, SCAD patients with a high MPO concentration > 300 ng/ml and a high concentration of inflammatory markers and reduced concentration of apoAI and HDL-C in which LpPLA2 was increased are at the risk of ACS and stroke [18–20]. Our studies have shown elevated levels of TG, apoB, and apoB/apoAI, TG/HDL-C ratios, and indicated an increasing concentration of triglyceride-rich lipoproteins (apoB-containing lipoproteins). TRLs caused an increase in the concentration of inflammatory markers and MPO levels in the artery wall [8, 9, 19–21]. These results suggest that a high MPO and TG level and disorder of apoB/apoAI and TG/HDL-C ratios together with high inflammatory markers decreased HDL-C and apoAI concentration and disturbed the composition of HDL-C and apoAI in HDL, and the anti-inflammatory and anti-oxidative functions of HDL particles [7, 13]. Khine HW et al. [5] showed that increased MPO indexed to HDL particle concentration (MPO/HDLp) is associated with increased risk of incident CVD (cardiovascular disease) events in a population initially free of CVD. MPO may contribute to atherosclerotic plaque instability [22].

Our results for the first time indicated that moderate dyslipidemia and dyslipoproteinemia caused chronic inflammation in artery wall and slowly increased the concentration of MPO, inflammatory markers and MPO/apoAI, MPO/HDL-C ratios and together gradually deterioration of dyslipidemia and dyslipoproteinemia. We also showed for the first time that the main problem of SCAD patients is not the high concentration of total cholesterol and LDL-C but the progressive increase chronic inflammation and the accompanying increase in TG and apoB-containing lipoprotein concentration, gradually and slowly decline of HDL-C, apo AI and lowering the HDL particles function. The rapid increase in systemic inflammation may cause an increase in MPO concentration which may cause ACS and stroke in patients who had low lipid and lipoprotein at the beginning of studies [5]. In the future, prospective studies of SCAD patients are needed, in groups with moderate and advanced inflammation which markers of inflammation, lipids, apoB-containing lipoproteins and HDL particles will be determined. Understanding the relationship between inflammation of apoB-containing lipoproteins and HDL particles will contribute to the implementation of effective, causal treatment of patients with SCAD [23, 24].

## Conclusion

The results suggest that moderate dyslipidemia and dyslipoproteinemia deepening of inflammation and inflammation slowly induce increase MPO concentration which decrease apoAI and HDL-C level and disturb HDLs function. The increasing MPO level and MPO/HDL-C, MPO/apoAI ratios can differentiate the SCAD of patients at the risk of acute coronary syndrome (ACAD) and stroke.

## Abbreviations

ACS: Acute coronary syndromes
HDL-C: High density lipoprotein – cholesterol
HOSCN: Hypothiocyanate
hsCRP: High sensitivity C-reactive protein
IL-6: Interleukine-6
LDL-C: Low density lipoprotein cholesterol
LpPLA2: Lipoprotein associated phospholipase A2
MPO: Myeloperoxidase
non-HDL –C: Non high density lipoprotein cholesterol
PON 1: Paraoxonase 1
SCAD: Stable coronary artery disease
TC: Total cholesterol
TNF-α: Tumor necrosis factor-α
